# Preparation and Performance Study of SiC-Reinforced Fe-Based Wear-Resistant Composite Grinding Media

**DOI:** 10.3390/ma17122940

**Published:** 2024-06-15

**Authors:** Kun Yang, Yongmin Zhou

**Affiliations:** College of Materials Science and Engineering, Nanjing Tech University, South Puzhu Road No. 30, Nanjing 211816, China; 202161203288@njtech.edu.cn

**Keywords:** metal matrix ceramic composites, powder metallurgy, grinding media, wear resistance, grinding efficiency

## Abstract

During industrial and laboratory processes involving material grinding, the grinding media endure prolonged high-collision and friction environments, resulting in substantial wear. Consequently, this study adopts the hot-pressing sintering technique in powder metallurgy to prepare SiC-reinforced Fe-based wear-resistant composite grinding media, aiming to increase wear performance. For this purpose, Fe with 10 wt% SiC powders were milled for the fabrication of the composite. Then, sintering was performed by hot press at 1100 °C in a furnace. Scanning electron microscopy (SEM) and X-ray diffraction were employed to investigate the microstructures and phase of SiC-reinforced Fe-based matrix composite. Subsequently, comparative performance evaluations of the newly developed grinding media and traditional chromium-based media were conducted in terms of wear rate and grinding efficiency. The wear resistance tests revealed that the SiC-reinforced composite media displayed significantly superior wear resistance across various abrasives compared to the chromium-containing alternatives. Specifically, the composite media achieved a wear rate reduction of 2.9 times against standard sand over 1 h, and 2.3 and 2.4 times against sandstone and iron slag, respectively. Moreover, extended grinding for 3 hours further enhanced these reductions to 3.1, 2.4, and 2.7 times, respectively. Additionally, efficiency assessments indicated that at a 1:1 material ratio, the composite media outperformed the chromium-containing media in grinding efficiency by 7.5%, 12.5%, and 10.3% for standard sand, sandstone, and iron slag, respectively. Further increasing the material ratio to 3:1 resulted in efficiency improvements of 7.4%, 17.5%, and 11.3%, correspondingly.

## 1. Introduction

Grinding media play a crucial role in the comminution of materials, and are extensively utilized in foundational industries such as cement, mineral processing, and metallurgy [1]. Selecting suitable grinding media is of paramount importance, not only because their consumption represents a significant portion of the operational costs in grinding processes, but also because they directly impact the grinding efficiency and the fineness of the final abrasives. Grinding media are widely adopted in various materials including iron, steel, and ceramics [2,3]. These grinding media exhibit considerable diversity in terms of both their materials and their geometric shapes. These shapes include such as spherical, rod-like (segments), short cylindrical, and truncated conical forms [4,5]. Additionally, the sizes of these grinding media also differ, with each shape designed to fulfill specific grinding conditions and requirements [6]. During the normal operation of a grinding mill, the grinding media are lifted to a certain height by centrifugal and inertial forces and then fall due to their own gravity, impacting and pulverizing the abrasives. During the impact process of the grinding media, the materials are continuously subjected to damage and fractures, forming new surface energy [7,8]. This also makes existing steel grinding media prone to wear, leading to failure and consequently increasing grinding costs. Generally, with regard to wear, surface hardness is an important aspect of wear resistance. Within the same category of materials, particularly in the absence of hard phases, higher hardness results in better wear resistance [9,10], although this may change as stress levels increase and toughness becomes increasingly important [11]. Several authors [12] indicate that the wear resistance of ductile materials and metal matrix composites (MMCs) is significantly enhanced. Therefore, using metal matrix composites to replace existing steel materials in the manufacture of grinding media can greatly improve their wear resistance. This is beneficial in addressing the issue of easy wear in existing grinding media.

Metal matrix composites (MMCs) are composed of a metal matrix [13] (typically Fe, Al, Cu, Mg, Ni, or Ti) and a reinforcing phase [14] (commonly nitrides, metal oxides, or carbides). The reinforcing phases, primarily in the form of particles or fibers, are selected to enhance the wear resistance of the composites. Hard and wear-resistant materials such as SiC and Al_2_O_3_ are selected for the reinforcement. These metal matrix wear-resistant composites exhibit a number of advantageous properties, including high hardness, good toughness, strong wear resistance, corrosion resistance, and low thermal expansion coefficient [15]. As a matrix material, iron (Fe) is in a central position in the industrial and construction sectors due to its abundance, low cost, and recyclability. The refining of iron is natural and environmentally friendly. Iron possesses high strength, excellent toughness, and superior wear resistance, rendering it an ideal foundational material for wear-resistant composites. These attributes enhance the performance of the composites while also delivering economic and environmental benefits. Silicon carbide (SiC), also known as refractory sand or carborundum, is a material of significant interest in the field of composite materials. Its high hardness, high wear resistance, and high temperature stability make it a popular choice for reinforcing particles [16,17]. Despite the numerous advantageous properties of SiC, which has become a crucial reinforcing particle in metal matrix composites, the current SiC-reinforced Fe-based wear-resistant composites encounter challenges such as fragile interfacial bonding [18]. The main reason for this discrepancy is the physical and chemical dissimilarities between the two materials, which hinder the performance development of wear-resistant composites. The fabrication of wear-resistant composites requires sintering at approximately 1000 °C. Due to the disparate thermal expansion coefficients of SiC and Fe, SiC shrinks less than Fe. This difference in shrinkage during the cooling process of the composite leads to the occurrence of residual stresses, causing defects at the interface and resulting in degraded performance [19]. The thermodynamic disadvantage of the SiC/Fe system was that SiC, used as a reinforcing particle in Fe and its alloys, reacts during the sintering process to form ferrosilicon compounds, such as FeSi, Fe_2_Si, and Fe_5_Si_3_ [20]. The formation of these ferrosilicon compounds can accumulate at the interface and lead to the deterioration of the microstructure and properties of the wear-resistant composite. However, minor chemical reactions do not affect the performance of SiC-reinforced Fe-based wear-resistant composite. Thus, controlling the extent of these chemical reactions is crucial in determining the interfacial bonding capability between the matrix and the reinforcing particles. Effective control of the chemical reactions requires research into the preparation processes of the composite.

The basic steps and key technologies of powder metallurgy begin with the preparation of the powders, which involves the mechanical alloying of different metal or alloy powders by high-energy ball milling [21,22] or ultrasonic pulverization methods [23]. This process can be used to produce alloy powders or mixed powders of different materials. Various powder materials (such as metal powders, additives, and reinforcing powders) are uniformly mixed to achieve the desired powder fineness. During the ball milling mixing process, grinding balls made of materials such as steel or zirconia are used [24]. several scientific studies have indicated that increasing the rotational speed of the ball mill and extending the duration of ball milling during the powder mixture process improve the properties of the prepared composite [25,26]. The powder mixture is then compacted under pressure into preformed shapes and sizes, a step that is usually performed using molds. Sintering is one of the most critical steps in the powder metallurgy process, this can involve atmospheric pressure sintering, hot pressing sintering [27], and plasma sintering techniques [28]. In this step, the pressed green body is heated to a temperature close to its melting point, but not to the point of melting. During the heating process, Sintering occurs between powder particles, leading to an increase in the density and strength of the material [29,30]. After sintering, the finished materials may need to undergo a series of post-processing steps such as heat treatment, surface treatment, etc. [31]. To meet the requirements of the final application. The powder metallurgy process offers the advantage of controlling the content and granulometry of the reinforcing phase during the mixing and grinding process and shows good application prospects in the preparation of high-performance materials and the development of new materials. Joshua Pelleg [32] used hot isostatic pressing and conventional sintering methods to prepare the Fe-SiC composite. The results show that there was no significant interfacial reaction under conventional sintering at 1173 K, 150 MPa for 2 h, but a small amount of interfacial reaction occurred at 1273 K. The formed reaction layer consisted of Fe_3_Si, FeSi, and FeSi_2_. The presence of these reaction layers could act as a diffusion barrier to prevent further decomposition of the SiC fibers, and it was observed that when the SiC mass fraction was 3%, Young’s modulus and tensile strength of the composite increased by 12.6% and 33.1%, respectively. Abenojar J and others [33] studied the effects of the type of reinforcement, addition amount, and sintering atmosphere on the properties of powder metallurgy 316L stainless steel during the sintering process. Commonly used reinforcement materials include AlCr_2_, Cr_2_Ti, and SiC, with volumes of 1.5% and 3%. All materials were mixed with 0.6% wax, compacted under 700 MPa, and sintered at 1230 °C. To analyze the sintering atmosphere, 3 different atmospheres were employed: 75% H_2_–25% N_2_, pure H_2_, and vacuum. The results indicate that SiC composite exhibited enhanced wear resistance, particularly when 3% SiC is incorporated and sintered in vacuum or hydrogen atmospheres. Wubin J [18] used conventional sintering methods to produce Cu-adhered SiC particles(Cu/SiC). The properties test results indicated that Cu/SiC particles reinforced with Fe matrix composites had better properties. Furthermore, the hardness and impact toughness improved up to 239.97 HV0.5 and 12.1 KJ·m^−2^, respectively. Moreover, compared to raw SiC particles, the hardness and impact toughness increased by 12% and 15%, respectively. Azam et al. [34] successfully designed and fabricated (Fe, SiC, Cu, and C) MMC, a composite sample with Fe (66.5%), Cu (11%), SiC (10% in the range of 45–150 um), graphite (6.5%), and BaSO4 (6%) was fabricated by hot press at 1000 °C in a controlled atmosphere furnace. In the pin-on-disc test, this sample had less weight loss than other samples. The total weight change in the distance of 1000 m is 0.0169 g. In the COF test, this sample had less fluctuation, and the graph seemed to stabilize at 0.35 after a certain distance. Consequently, this sample will have more wear resistance and can meet expectations with 10% SiC for aircraft brake applications.

Therefore, this study demonstrates that using the hot-pressing sintering method in powder metallurgy can be employed to prepare SiC-reinforced Fe-based wear-resistant composite grinding media, with the objective of reducing their wear rate. This is of significant importance for enhancing economic benefits. This study focuses on the preparation of grinding media using a single type of SiC-reinforced Fe-based wear-resistant composite material. It investigates the effects of four factors—SiC content, SiC particle size, sintering temperature, and holding time—on the wear rate of the grinding media and their efficiency in grinding materials. An orthogonal experiment design was employed to conduct multi-factor and multi-level experiments with the objective of exploring the optimal process route for preparing the grinding media. Subsequently, the phase composition and microstructure of the grinding media were analyzed and discussed. Finally, the performance of the grinding media prepared using SiC-reinforced Fe-based wear-resistant composite materials in this study was compared with that of the chromium-containing grinding media currently used in mills, to verify the rationality of the grinding media prepared in this study.

## 2. Materials and Methods

### 2.1. Raw Materials and Abrasives

**Figure 1:**
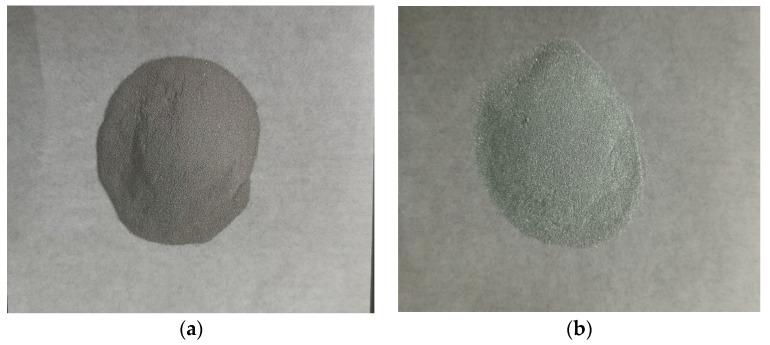
(**a**) Fe powder (**b**) SiC powder.

**Figure 2:**
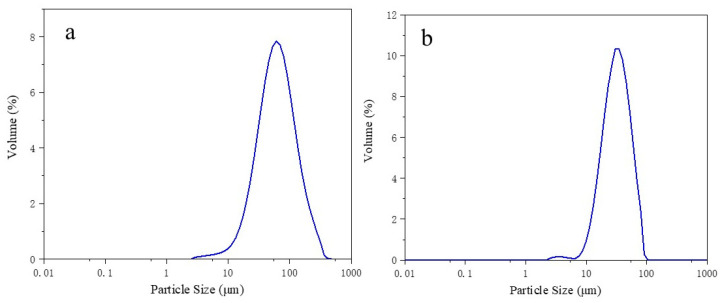
The particle size distribution of the two raw materials (**a**) Fe powder (**b**) SiC powder.

In this study, the reinforcing phase material utilized was green silicon carbide powders (produced by Dongtai Aidong Abrasive Mould Factory, Dongtai, China), which exhibited a higher degree of purity (98.85%) and a particle size range of 2.188–91.201 μm. The matrix material selected was reduced iron powders (produced by Sinopharm Chemical Reagent Co., Ltd., Shanghai, China) with a purity of 98.50% and a particle size range of 2.512–363.078 μm. Figure 1 presents images of the two raw materials, Figure 2 shows the particle size distribution of the two raw materials, and Table 1 and Table 2 list the chemical compositions of the two powders.

**Figure 3:**
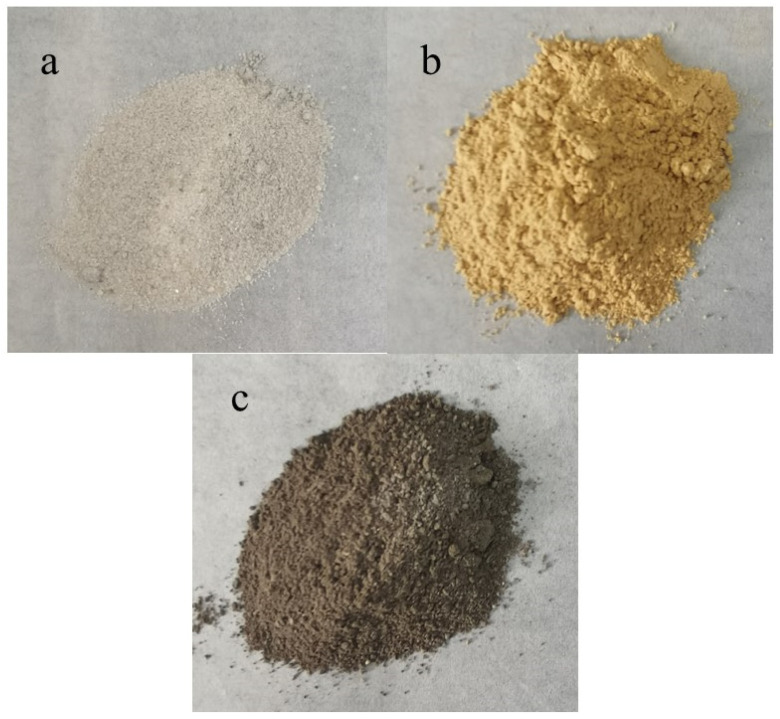
Images of the three types of sieved abrasives (**a**) standard sand, (**b**) sandstone, (**c**) iron slag.

The abrasives utilized were standard sand, sandstone, and iron slag. Before measuring the grinding efficiency, it was imperative to sieve through a 0.9 mm mesh to screen out larger particles, thus preventing significant errors in measuring the specific surface area of the powder. There was no necessity to sieve the abrasives when measuring the wear rate of the grinding media. Figure 3 shows images of the three types of sieved abrasives. The chromium-containing grinding media (forged steel with a Cr content of 1–3%) are of identical size to the prepared grinding media.

### 2.2. Orthogonal Experiment for the Preparation of Composite Material Grinding Media

**Figure 4:**
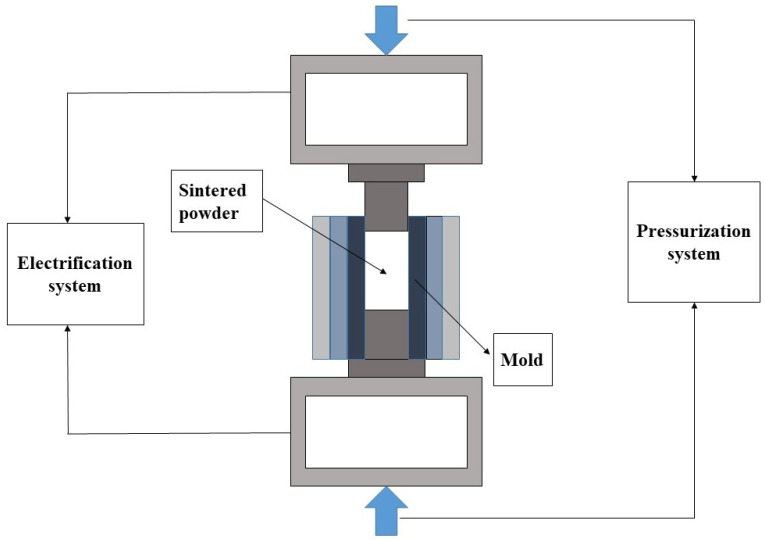
Simplified diagram of a hot press sintering apparatus.

(1) A specific ratio of reduced Fe powder and SiC powder was combined. The preparation of the powder mixture involves using wet ball milling to prevent the oxidation of the Fe powder during grinding. For the grinding and mixing media, 304 stainless steel balls were used. Each milling jar contained 100 mL of anhydrous ethanol, with a grading of 10 large balls with a diameter of 20 mm and 20 small balls with a diameter of 10 mm, at a material ratio of 1:2 and a rotation speed of 200 rpm. The mixing time was set. Subsequently, the powder mixture was subjected to vacuum drying for future use.
(2) Preparation of grinding media by hot pressing sintering: In this experiment, conductive hot pressing sintering methods and equipment were employed to prepare the grinding media. The powder mixture was placed in a mold and pressed into a cylinder with a diameter of approximately 20 mm and a height of 25 mm. Figure 4 is a simplified diagram of the hot-pressing sintering process. The molding pressure was set to 10 MPa. To enhance the performance of the grinding media, an orthogonal experiment was conducted with a four-factor, three-level experimental design for process research. The four factors considered in the study were the particle size of the powder mixture (grinding time), the content of the SiC, the temperature of the sintering process, and the holding time. The objective is to optimize the wear rate of the grinding media and the efficiency of grinding abrasives. Table 3 shows the design of influencing factors and levels.

### 2.3. Characterization of Composite

The microstructure of Fe/SiC particles and the interface reaction were observed by scanning electron microscopy (SEM, TM3000, HITACHI, Tokyo, Japan). The phase composition of the composite was determined by X-ray diffraction (XRD) with Cu-Kα radiation (SmartLab, Rigaku Corporation, Tokyo, Japan, 3 KW), at a scanning speed of 10° min^−1^. The particle size of all powders was measured using a laser particle size analyzer (Mastersizer 2000, Malvery Instruments Ltd., Malver, Britain). This paper employs the Brunauer–Emmett–Teller (FBT-9 FULL AUTOMATION INSTRUMENT TESTING SPECIFIC SURFACE, Zhongke Lanjian Instrument Equipment Co., Ltd., Beijing, China) method to measure the specific surface area of the abrasives.

### 2.4. Measurement of Grinding Media Performance

(1) Wear resistance measurement

**Figure 5:**
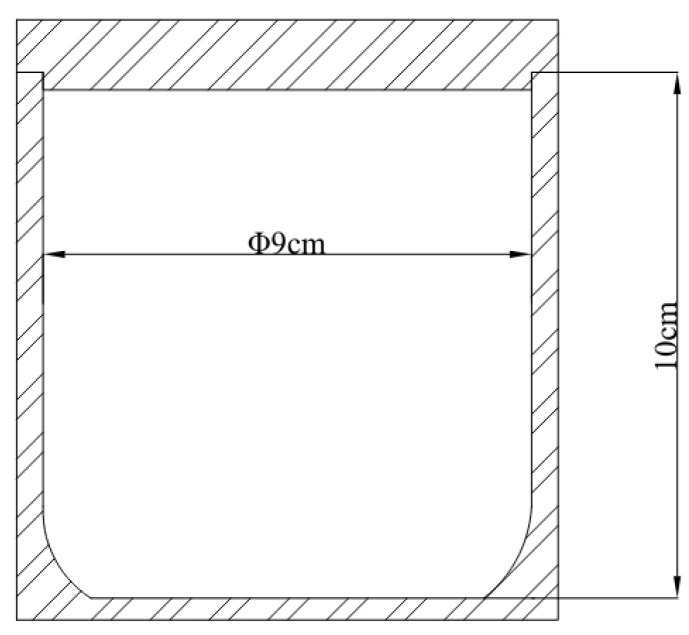
Diagram of the ball mill jar dimensions.

First, calculate the mass of the material to be added based on the material ratio (the mass of the grinding media to the mass of the abrasives added to the ball mill). Set the grinding speed at 200 rpm and the grinding time, replace the abrasives every 20 min of grinding. Next, after each grinding session, clean, dry (at 100 °C for 30 min), weigh, and record the weight of the grinding media. Figure 5 depicts the dimensions of the ball mill jar.

Then, calculate the wear rate using Equation (1):

(1) $$Z=\frac{m_{1}-m_{2}}{m_{1}}\times 100\%,$$

In the equation: *Z* represents the wear rate, *m*_1_ is the mass of the grinding media prior to grinding, and *m*_2_ is the mass of the grinding media following grinding.

(2) Grinding abrasives efficiency

The grinding efficiency ratio is defined as the ratio of useful work performed per unit time during the grinding process to the power consumption for grinding. The assessment method is proposed as follows in Equation (2):

(2) $$\eta =\frac{W\times h}{E},$$

In the equation, *η* represents the grinding efficiency, *W* is the useful work carried out during the grinding process, *H* is the time taken for grinding, and *E* is the power consumption during the specified time for grinding.

As the powder particles undergo continuous reduction in size during the grinding process, their surface energy increases. This increase in energy represents the useful work carried out during grinding. Therefore, Equation (2) can be rewritten as the following Equation (3):

(3) $$\eta =\frac{\sigma }{E},$$

In the equation, *σ* represents the increment in surface energy of the abrasives during the grinding process.

So, the increment in surface energy can be represented by the following Equation (4):

(4) $$\sigma =(S_{2}-S_{1})\sigma_{0},$$

In the equation, *S*_2_ represents the surface area after grinding, *S*_1_ denotes the surface area before grinding, *σ*_0_ is defined as the surface energy per unit area.

The following Equation (5) represents the equation for calculating specific surface area, which can be employed for the purpose of convenient testing in order to deduce surface area changes:

(5) $$\sigma =(S_{5}-S_{4})m\sigma_{0}$$

In the equation, *S*_5_ represents the specific surface area of the measured abrasives after grinding while *S*_4_ represents the specific surface area of the measured abrasives before grinding, and *m* denotes the mass of the measured abrasives.

The derivation from the above equations indicates that the preparation of grinding media requires comparison with chromium-containing grinding media for their grinding efficiency. Therefore, measuring the specific surface area of abrasives before and after grinding is sufficient to determine the grinding efficiency.

## 3. Results and Discussion

### 3.1. Analysis of Orthogonal Experiment Results

Table 4 presents the L9(3^4) orthogonal experiment table along with the corresponding performance results. The wear rate measurement conditions are as follows: grinding time of 1 h, ball mill speed of 200 rpm, grinding with standard sand, and a material ratio of 1:2. The conditions for measuring grinding efficiency are as follows: the grinding time for standard sand was 10 min, the ball mill speed was 200 rpm, and the material ratio was 1:2. Table 5 presents the sum of the wear rates for each level of the factors, represented by K_1_, K_2_, and K_3_. The average wear rates at each level are represented by k_1_, k_2_, and k_3_. The results of the range analysis indicate that the grinding time of the powder mixture (particle size) has the largest range, 0.22, followed by content at 0.11, a holding time at 0.06, and the smallest range is a temperature of 0.05. This demonstrates that among the four factors, the powder mixture grinding time (particle size) has the greatest impact on the wear rate of the grinding media, followed by SiC content, then the holding time, with temperature having the least impact. The results of the range analysis indicate that, from the perspective of improving the wear rate of grinding media through the preparation process, the optimal preparation should involve the following variables: a grinding time of the powder mixture of five hours, incorporating 10 wt% SiC, sintering at 1100 °C, and a holding time of 15 min. 

Table 6 presents the sum of the changes in specific surface area for each level of the factors, represented by K_1_, K_2_, and K_3_. The average changes in specific surface area at each level are represented by k_1_, k_2_, and k_3_. The results of the range analysis indicate that the content has the greatest range at 6.37, followed by holding time at 5.77, and the grinding time of the powder mixture (particle size) at 5.03. Temperature has the smallest range at 3.46. These findings indicate that among these four factors, the SiC content has the greatest impact on the grinding efficiency of the grinding media, followed by holding time, then the powder mixture grinding time (particle size), with temperature having the least impact. The results of the range analysis indicate that, from the perspective of improving the abrasives grinding efficiency of the grinding media through the preparation process, the optimal preparation should involve the following parameters: a grinding time of the powder mixture of ten hours, incorporating 20 wt% SiC, sintering at 1100 °C, and a holding time of 10 min. In conclusion, from the perspective of the wear resistance of grinding media, the larger the SiC particle size, the lower the wear rate of the prepared grinding media. This is due to the fact that larger reinforcing particles in the powder can withstand greater impact forces, thereby making the matrix less susceptible to wear, and the grinding efficiency is also satisfactory. Furthermore, to enhance the density of the grinding media and improve their overall performance, particles of an appropriate size should be selected. The determined process parameters are as follows: a grinding time of the powder mixture of five hours, incorporating 10 wt% SiC, sintering at 1100 °C, and a holding time of 15 min.

### 3.2. XRD and SEM Analysis Results

**Figure 6:**
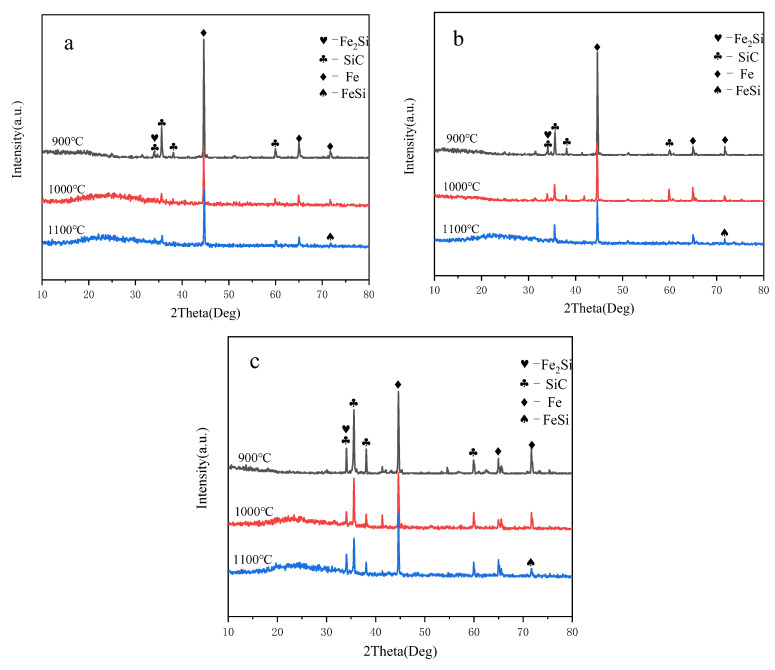
XRD patterns of samples with SiC to Fe mass ratios of (**a**) 1:9, (**b**) 1:4, and (**c**) 3:7 after holding at 900 °C, 1000 °C, and 1100 °C for 15 min, respectively.

As illustrated in Figure 6, the three distinct proportions exhibit a consistent trend at their respective sintering temperatures. At sintering temperatures of 900 °C and 1000 °C, due to the relatively low reaction temperatures, the chemical reaction between SiC and Fe is just beginning. This results in the emergence of highly pronounced diffraction peaks for both SiC and Fe and a slight diffraction peak of Fe_2_Si can be observed. Conversely, the diffraction peak of FeSi does not manifest. As the sintering temperature is elevated to 1100 °C, the reaction rate accelerates, resulting in a more rapid chemical reaction between the two raw materials compared to the previous sintering temperatures. Consequently, the intensity of the diffraction peaks for SiC and Fe decreases further. Not only does the diffraction peak of Fe_2_Si appear, but also the diffraction peak of FeSi becomes evident. However, both peaks are less pronounced compared to the original materials peaks, indicating that the extent of the reaction at this temperature is minimal. The preceding analysis leads to the conclusion that a significant acceleration of the chemical reaction rate between SiC and Fe is achieved by increasing the sintering temperature. This promotes the occurrence of solid-phase reactions. At a sintering temperature of 1100 °C, the diffraction peaks of Fe_2_Si and FeSi are present but not pronounced. This indicates that the reaction between SiC and Fe at this temperature is relatively mild and more amenable to process control.

**Figure 7:**
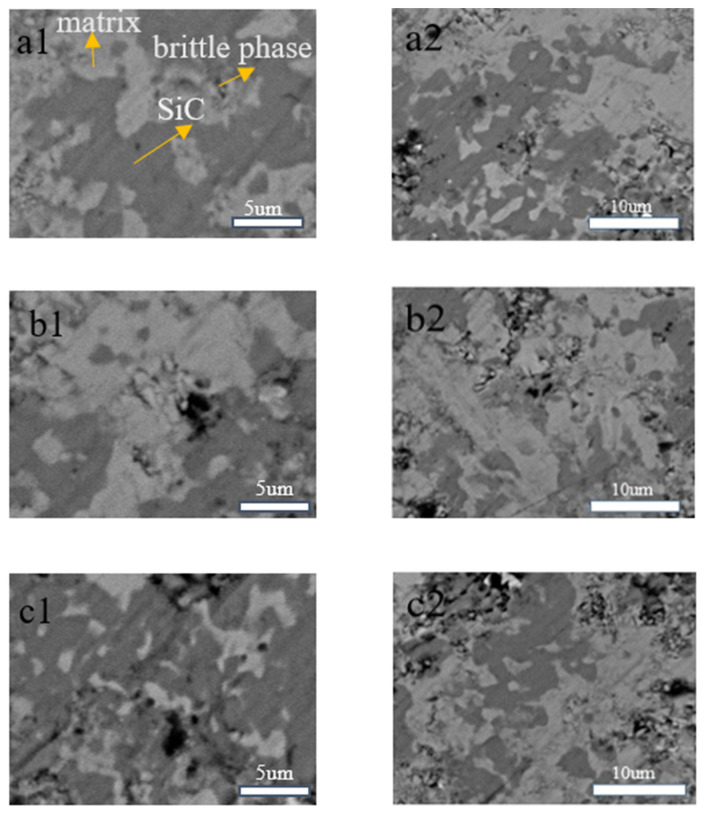
SEM images of samples with SiC to Fe mass ratios of (**a1**,**a2**) 1:9, (**b1**,**b2**) 1:4, and (**c1**,**c2**) 3:7 after holding at 1100 °C for 15 min.

Figure 7 illustrates that the bright white areas represent the Fe matrix, the gray areas are SiC reinforcing particles, and the black areas are the product carbon of the reaction. As the SiC content increases, the area of the black region has a trend of increasing. This phenomenon indicates that an elevated SiC content results in more frequent and intense reactions with Fe, thereby leading to the generation of greater quantities of ferrosilicon compounds and carbon. These reaction products tend to accumulate in the interfacial bonding areas of the composite, which may affect the overall performance of the wear-resistant composite. This phenomenon can be observed in the case of a Fe matrix, where the addition of SiC increases its wear rate due to the higher wear resistance of SiC compared to Fe. When the grinding media is mixed with abrasives for grinding, the Fe matrix, which has a lower wear resistance than SiC particles, tends to concave, causing the SiC particles to protrude. At this point, the SiC particles primarily bear the wear, acting to protect the matrix from abrasion, thereby further reducing the wear of the matrix. Indeed, as the SiC particle content increases, the particles are prone to detachment during grinding, which can lead to an increase rather than a decrease in wear. This occurs because the density and toughness of the Fe-based wear-resistant composite decrease, and the porosity increases. During the friction process, the number of effective particles bearing the load is reduced, resulting in the protruding SiC particles bearing greater forces. Since the bonding between SiC particles and the Fe matrix may not be strong enough, these particles are more prone to detachment from the surface of the matrix under significant pressure. Once these hard SiC particles fall off, they expose new wear surfaces, thereby accelerating the wear process and leading to a continuous increase in wear rate.

### 3.3. Comparative Study of Grinding Media Performance

Table 7 presents the density measurements for different abrasives. The densities of standard sand, sandstone, and iron slag are 2627.4, 2903.5, and 3040.4, respectively.

**Figure 8:**
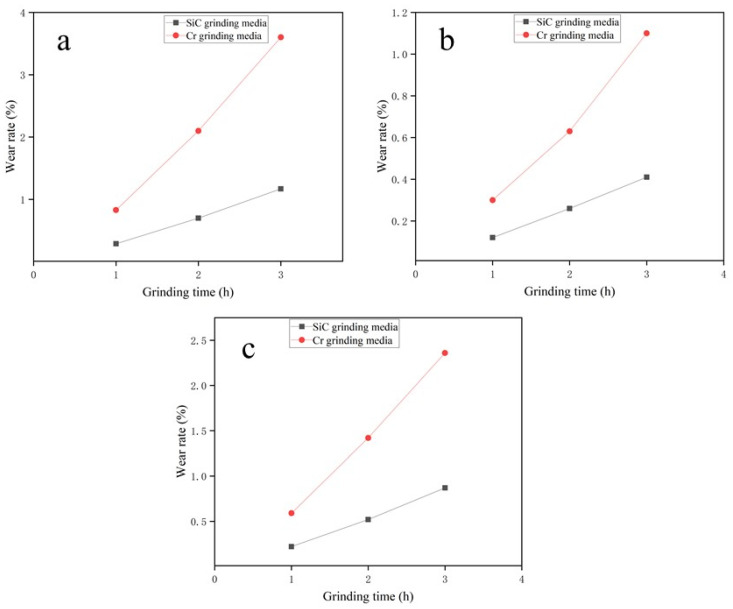
Graph showing the relationship between wear rate and grinding time for grinding different abrasives with the parameters of 200 rpm and powder-to-material ratio of 1:1 (**a**) standard sand, (**b**) sandstone, (**c**) iron slag.

Figure 8 presents a comparative analysis of the wear rates of grinding media as the grinding time increases. The graph includes two variables: grinding time measured in hours and wear rate percentage. Specifically, grinding media manufactured from SiC-reinforced Fe-based wear-resistant composite exhibit a more gradual increase in wear over time, accompanied by varying degrees of wear rate reduction for different abrasives. When the grinding time is 1 h, the wear rate of grinding media made from SiC-reinforced Fe-based wear-resistant composite is reduced the most when grinding standard sand, achieving a reduction of 2.9 times while the reduction in wear rate is slightly lower when grinding sandstone and iron slag, at 2.3 times and 2.4 times, respectively. When the grinding time is extended to 3 h, the multiple of wear rate reduction when grinding standard sand increases to 3.1 times, and for grinding sandstone and iron slag, the reductions are 2.4 times and 2.7 times, respectively. The analysis indicates that grinding media made from SiC-reinforced Fe-based wear-resistant composite exhibit significantly superior wear resistance compared to those containing chromium. During a continuous 3 h grinding process, grinding media made from SiC-reinforced Fe-based wear-resistant composite demonstrate more stable wear resistance, with a more gradual increase in wear rate. This result may be attributed to the physical properties of SiC, such as its excellent wear resistance. Conversely, the rapid increase in the wear rate of chromium-containing grinding media may indicate their lower stability under continuous friction and impact. In long-term grinding of abrasives, the use of grinding media made from SiC-reinforced Fe-based wear-resistant composite offers better durability and cost-effectiveness.

**Figure 9:**
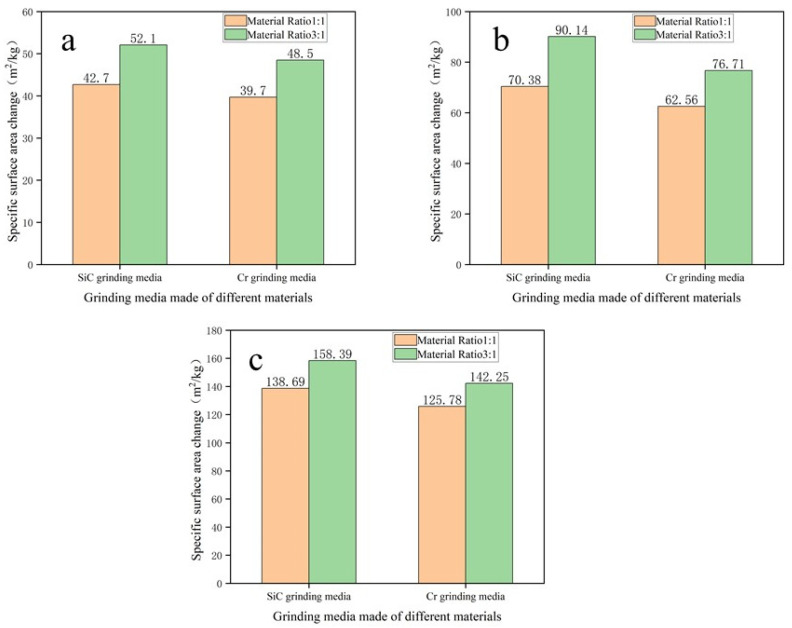
Relationship between different material ratios and the amount of change in the specific surface area of abrasives with the parameters of 200 rpm and milling time for 10 min (**a**) standard sand, (**b**) sandstone, (**c**) iron slag.

**Figure 10:**
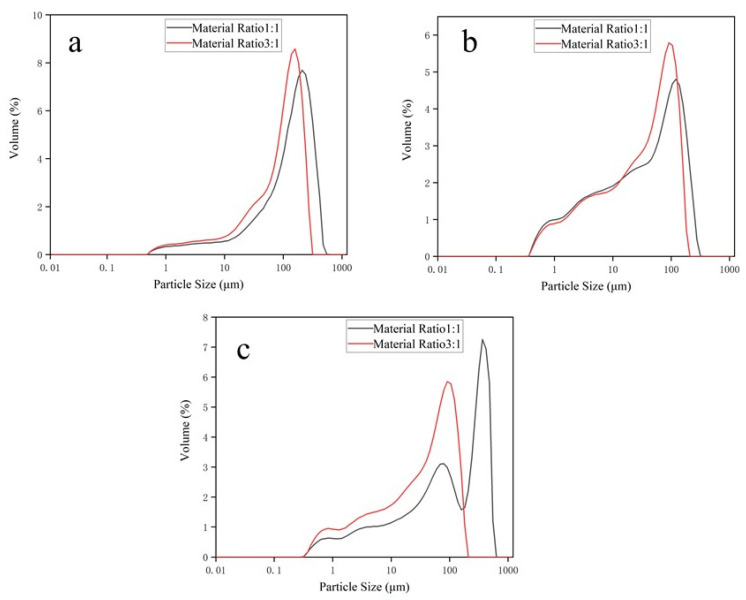
Particle size distribution graph for grinding abrasives with composite grinding media at different material ratios with the parameters of 200 rpm milling time for 10 min (**a**) standard sand, (**b**) sandstone, (**c**) iron slag.

Figure 9 displays the changes in specific surface area for two types of grinding media made from two different materials (SiC and Cr) under different material ratios. In Figure 9, the orange line represents a material ratio of 1:1, while the green line represents a material ratio of 3:1. When the material ratio is 1:1, the grinding efficiency of grinding media made from SiC-reinforced Fe-based wear-resistant composite compared to those containing chromium for grinding standard sand, sandstone, and iron slag increased by 7.5%, 12.5%, and 10.3%, respectively. When the material ratio is 3:1, the grinding efficiency of grinding media made from SiC-reinforced Fe-based wear-resistant composite compared to those containing chromium for grinding standard sand, sandstone, and iron slag increased by 7.4%, 17.5%, and 11.3%, respectively. This phenomenon indicates that for both SiC-containing and chromium-containing grinding media, an increase in material ratio enhances grinding efficiency. This is because a higher material ratio allows more abrasives to participate in the grinding process within a set time, thereby improving the grinding effect. Additionally, grinding media manufactured from SiC-reinforced Fe-based wear-resistant composite show a higher change in specific surface area under both material ratio conditions compared to those containing chromium, indicating that SiC grinding media exhibit superior grinding efficiency. Figure 10 presents a particle size distribution graph for abrasive particles, illustrating the relationship between particle distribution and particle size for varying material ratios. The horizontal axis represents particle size (in microns) on a logarithmic scale, while the vertical axis represents the percentage of particle size distribution. Figure 10 illustrates the particle size distribution at two different material ratios: 1:1 (represented by the gray line) and 3:1 (represented by the red line). At the 1:1 ratio, the particle size distribution exhibited a somewhat broader range, while at the 3:1 ratio, the distribution exhibited a more pronounced and elevated peak, indicating a more concentrated particle size distribution. Furthermore, the peak of the red line appears to the right of the peak of the gray line, indicating that the average particle size is smaller when the material ratio is 3:1 compared to 1:1. This suggests that a higher material ratio results in a smaller average particle size of the ground material.

## 4. Conclusions

(1) An orthogonal experiment was employed to investigate the relationship between the wear rate of grinding media and the efficiency of grinding abrasives. The factors selected for exploration included the grinding time of the powder mixture, SiC content, sintering temperature, and holding time. The optimal preparation process was identified as follows: the grinding time of the powder mixture was five hours, incorporating 10 wt% SiC, sintering at 1100 °C, and a holding time of 15 min.
(2) The phase changes in the sintered grinding media were analyzed using XRD technology. As the sintering temperature increased, the diffraction peak of Fe_2_Si appeared and intensified. By 1100 °C, the diffraction peak of FeSi also began to appear; however, these peaks were relatively weak compared to the raw materials, indicating that the reactions at these temperatures were not very intense.

The application of SEM technology enabled the identification of the microstructure of the sintered grinding media. The white regions correspond to the Fe matrix, which provides structural support whereas the gray areas represent the wear-resistant SiC-reinforcing particles. The black regions denote the presence of carbon, formed during the sintering process.

(3) The results of the wear rate tests on grinding media indicate that those made from SiC-reinforced Fe-based wear-resistant composite had a lower wear rate compared to those containing chromium, especially when grinding standard sand. After 1 h of grinding, the wear rate decreased by 2.9 times, and after 3 h, it decreased by 3.1 times. Although the reduction is slightly less for sandstone and iron slag, it remains significant.

Regarding grinding efficiency, media made from SiC-reinforced Fe-based wear-resistant composites demonstrated improved efficiency compared to those containing chromium when grinding various abrasives. The efficiency increases by 7.5% for standard sand, 12.5% for sandstone, and 10.3% for iron slag at a material ratio of 1:1. At a material ratio of 3:1, the efficiency improvements are 7.4% for standard sand, 17.5% for sandstone, and 11.3% for iron slag. Additionally, higher material ratios result in finer particle sizes of the ground materials.

## Figures and Tables

**Table 1:** The chemical composition of Fe powder.

Chemical Composition	Fe	Al	Ca	Si	Mn	Others
Content (%)	98.5000	0.2600	0.1850	0.2560	0.4010	0.3980

**Table 2:** The chemical composition of SiC.

Chemical Composition	SiC	Ca	Ni	Fe	Al	Others
Content (%)	98.8500	0.0965	0.0414	0.4030	0.0224	0.5867

**Table 3:** The design of influencing factors and levels.

Serial Number	Grinding Time (h)	Content (wt%)	Temperature (°C)	Holding Time (min)
1	15	10	900	10
2	10	20	1000	15
3	5	30	1100	20

**Table 4:** Summary table of four-factor three-level orthogonal experiment results.

Serial Number	Factors				Wear Rate (%)	Change in SSA (m^2^/kg)
	Grinding Time (h)	Content (wt%)	Temperature (°C)	Holding Time (min)		
1	1	1	1	1	0.40	29.8
2	1	2	2	2	0.51	31.1
3	1	3	3	3	0.55	26.1
4	2	1	2	3	0.35	29.6
5	2	2	3	1	0.24	39.2
6	2	3	1	2	0.39	33.3
7	3	1	3	2	0.21	34.4
8	3	2	1	3	0.25	26.2
9	3	3	2	1	0.34	30.2

**Table 5:** Analysis results of the range of wear rate.

Serial Number	Factors			
	Grinding Time (h)	Content (wt%)	Temperature (°C)	Holding Time (min)
K_1_	1.46	0.96	1.04	0.98
K_2_	0.98	1.00	1.20	1.11
K_3_	0.80	1.28	1.13	1.15
k_1_	0.49	0.32	0.35	0.32
k_2_	0.33	0.33	0.40	0.37
k_3_	0.27	0.43	0.38	0.38
Range(R)	0.22	0.11	0.05	0.06

**Table 6:** Analysis results of the range of specific surface area change.

Serial Number	Factors			
	Grinding Time (h)	Content (wt%)	Temperature (°C)	Holding Time (min)
K_1_	87.0	89.6	89.3	99.2
K_2_	102.1	104.7	90.9	98.8
K_3_	90.8	85.6	99.7	81.9
k_1_	29.00	29.87	29.77	33.07
k_2_	34.03	34.90	30.30	32.93
k_3_	30.27	28.53	33.23	27.30
Range(R)	5.03	6.37	3.46	5.77

**Table 7:** Density measurement table for different abrasives.

	Measure Density Values(kg/m^3^)			
Abrasives	Number One	Number Two	Third	Average Value
Standard sand	2629.7	2596.3	2656.4	2627.4
Sandstone	2915.3	2867.6	2927.5	2903.5
Iron slag	3016.8	3125.6	2978.7	3040.4

## Data Availability

The original contributions presented in the study are included in the article, further inquiries can be directed to the corresponding author.
